# IGF-1 and Bone: New Discoveries From Mouse Models

**DOI:** 10.1002/jbmr.234

**Published:** 2010-08-23

**Authors:** Shoshana Yakar, Hayden-William Courtland, David Clemmons

**Affiliations:** 1 Division of Endocrinology, Diabetes and Bone Disease, Mount Sinai School of Medicine New York, NY, USA; 2 Division of Endocrinology, University of North Carolina Chapel Hill, NC, USA

**Keywords:** INSULIN-LIKE GROWTH FACTOR, GROWTH HORMONE, BONE ACCRUAL, SKELETAL GROWTH, CORTICAL-BONE

## Abstract

Insulin-like growth factor-1 (IGF-1) plays a central role in cellular growth, differentiation, survival, and cell cycle progression. It is expressed early during development and its effects are mediated through binding to a tyrosine kinase receptor, the insulin-like growth factor-1 receptor (IGF-1R). In the circulation, the IGFs bind to IGF-binding proteins (IGFBPs), which determine their bioavailability and regulate the interaction between the IGFs and IGF-1R. Studies in animal models and in humans have established critical roles for IGFs in skeletal growth and development. In this review we present new and old findings from mouse models of the IGF system and discuss their clinical relevance to normal and pathological skeletal physiology. © 2010 American Society for Bone and Mineral Research.

## Introduction

From *sulfation factor*, discovered in the 1950s, to *somatomedin* in the 1970s, to *insulin-like growth factor 1* (IGF-1) in the 1980s, we have learned that IGFs play a central role in growth, development, and metabolism. While originally IGFs were discovered in serum and found to be produced by the liver, numerous studies in the 1980s demonstrated that virtually all tissues express IGF-1. The recognition of IGF-1 as an endocrine (serum) and an autocrine/paracrine (tissue) hormone has helped the scientific community develop an understanding of how these two systems function coordinately. It has become clear that liver IGF-1 is regulated largely by the ambient levels of growth hormone (GH) secreted from the pituitary, but this is not necessarily the case for non–hepatic tissue production of IGF-1. Additionally, the intimate relationship between GH and IGF-1 has made it difficult to attribute discrete actions to each hormone, and in the past two decades, a great deal of effort has been made to unravel these functions. In this article we discuss the effects of GH/IGF-1 on the skeleton as derived from mouse models. The models are presented in the context of what they tell us about IGF-1's role as a global growth factor, an endocrine factor, and a tissue growth factor and are placed, where possible, in the context of human IGF-1 deficiencies.

## IGF as a Global Growth Factor

Given that the bulk of circulating IGF-1 is produced through GH action on the liver, it was expected that inhibition of GH signaling in mice would retard growth significantly. Indeed, in Snell dwarf (*dw*/*dw*) mice, deletion of the transcription factor gene *pit1* resulted in a loss of GH production(1) and reduced bone length owing to a reduction in cartilage hypertrophy and delayed epiphyseal ossification.(2–3) Such alterations are likely the mechanism behind marked growth retardation at 2 weeks of age and complete growth arrest at the onset of puberty (4 weeks of age).(4) Similar phenotypes were found in the Ames dwarf mouse (*dt*/*dt*), where *df* (prop-1, an upstream regulator of pit-1) is absent and GH is again not produced. Further, Ames dwarf male and female mice had decreases in body weight that were accompanied by reductions in lean mass, bone area, and bone mineral content (BMC).(5) It should be noted, however, that deficiencies in prolactin and thyroid-stimulating hormone (TSH) are also present in both Snell(6,7) and Ames(8) dwarf mice. Thus the aforementioned phenotypic changes may not be due entirely to GH effects. When *Stat5b*, a downstream effector of the GH receptor (GHR), was ablated in mice, body weights and bone lengths were reduced in a manner similar to Ames and Snell dwarf mice, although this effect was mainly apparent in male mice.(9) The defects of Snell and Ames dwarf as well as *Stat5b*^*−/−*^ mice resulted in blunted GHR activity and are similar to the clinical characteristics of Laron syndrome in humans, where the GHR is mutated. The clinical details of Laron syndrome are extensive and beyond the scope of this review, but they have been published previously.(10) These patients have low spinal and femoral neck areal bone mineral density (BMD) but volumetric BMD is normal. Bone size is markedly decreased compared with controls.(11) It should be noted that the *Stat5b*^*−/−*^ model has clinical significance of its own given that *Stat5b* mutations have been found in humans, and the phenotype of growth retardation is similar to that of *Stat5b*^*−/−*^ mice. However, the *Stat5b*^*−/−*^ mouse model has a sexually dimorphic phenotype (growth retardation is largely in males), whereas human cases of *Stat5b* deletions/mutations have demonstrated significant (and comparable) growth retardation in both females(12–14) and males.(15)

Total inactivation of the *Igf1* gene in mice resulted in 80% perinatal lethality. The surviving pups were 50% smaller than controls, highlighting the importance of Igf-1 in early growth.(16) This has been confirmed in studies of embryonic growth showing that fetal mice had short-limb dwarfism delays in mineralization and increased chrondrocyte apoptosis.(17) In *Igf1*^*−/+*^ mice it was reported that body weights, femur lengths, and BMD values were reduced in females and males, but the differences in males appeared only after puberty.(18) In terms of bone morphology, *Igf1*^*−/+*^ mice were found to have significantly reduced cortical area and periosteal circumference by 2 months (after puberty) in both males and females. This finding is supported by another study of *Igf1*^*−/+*^ mice that indicated that, at least in terms of transverse size (periosteal circumference), the differences appear as early as 3 weeks of age.(19) Bikle and colleagues reported that *Igf1*^*−/−*^ mice had a 24% reduction in cortical bone size and shortened femoral lengths, but trabecular bone density and connectivity were increased.(20) These investigators also demonstrated a defect in osteoclastogenesis and showed that osteoclasts were smaller with fewer nuclei in these knockouts. Additionally, expressions of RANKL and c-fms [the receptor for monocyte colony-stimulating factor (M-CSF)] were reduced significantly.(21) Interestingly, human patients with *IGF1* gene deletions have been identified. Patients homozygous for *IGF1* gene deletions exhibit growth retardation (short stature) and normal genitalia and proceed through puberty, but at a much slower rate.(22) Bone size is reduced at birth, and vertebral BMD was reduced, but this was due primarily to a decrease in bone volume.(23) IGF-1 replacement therapy had a significantly greater effect on bone volume than on bone density. Mice with deletion of the *Igf1* receptor (*Igf1r*^*−/−*^) resembled *Igf1* null mice in that pups were born smaller than controls (organ hypoplasia) and died shortly after birth.(24) In addition, the authors noted that primary ossification centers in the cranial and facial bones appeared later in *Igf1r*^*−/−*^ mice compared with controls. Studies of *Igf1r*^*+/−*^ mice found significant reductions in body weight by 4 weeks of age, although this was noticed only in males.(25) Although human patients with total *IGF1R* deletions have not been identified, patients with heterozygous mutations in the *IGF1R* receptor have been identified(26–29) and show various forms of intrauterine growth retardation and blunted postnatal growth,(30–34) as might be expected from studies of *Igf1r*^*−/−*^ mice. When the downstream effector of *Igf1r*, the insulin-receptor substrate 1 (Irs-1) was mutated in mice, significant reductions in body weight were apparent from birth and through adulthood.(35) Interestingly, these *Irs1*^*−/−*^ mice showed no delay in ossification of their long bones. In contrast, analysis of mice that had a spontaneous mutation in *Irs1* resulting in failure to translate the protein showed growth retardation, low bone mineral density, reduced cortical and trabecular thickness, and low bone-formation rates.(36) The human phenotype is similar to the latter model in that weight and body length are reduced at birth.(37)

Reduced Igf-1 bioavailability was demonstrated in transgenic mice with ubiquitous expression of Igf-binding proteins (Igfbps) and the acid-labile subunit (ALS). Overexpression of ALS resulted in reduced body weight gains during the first 3 weeks of growth and significantly reduced body weights through puberty.(38) Overexpression of Igfbp-1 resulted in growth retardation and a delay in mineralization of several bones (ie, craniofacial, metacarpal, and vertebral).(39) Igfbp-2 overexpression also resulted in growth retardation and mineralization defects. Specifically, whole-body BMC, femoral BMC, tibial BMC, and femoral volume were reduced in *Igfbp2* transgenics.(40) In *Igfbp3* transgenics, overexpression demonstrated a similar phenotype with decreases in body weight, cortical bone density, cortical bone volume, and cortical thickness.(41) These deficiencies were likely a result of a surface-specific cellular deficiency because bone-formation rates were significantly reduced on the periosteal surface. Further, cancellous bone density and trabecular thickness were decreased in *Igfbp3* transgenics. Similar changes in cortical and cancellous bone were found in *Igfbp5* transgenics, although it was reported that the skeletal phenotype was more severe in males than in females.(42) Endosteal bone formation was not inhibited but rather was enhanced as a result of Igfbp-5 overexpression. It should be noted that these *Igfbp* transgenics often do not alter total serum Igf-1 levels. However, in Igfbp overexpression models, although serum Igf-1 levels may be normal, the amount of extracellular fluid Igf-1 that is available to bind to tissue receptors is reduced, and this likely contributes to the observed bone and growth phenotypes.

In 1988, Mathews and colleagues created a transgenic mouse line expressing human IGF-1 in nearly all tissues.(43) During puberty (4 to 6 weeks of age), both female and male transgenic mice exhibited significant increases in body weight that lasted throughout the study (52 weeks of age). Numerous organ weights were found to be larger in mice overexpressing human IGF-1 (owing to hyperplasia), indicating that IGF-1 action from birth and through puberty leads to proportional size increases in a variety of tissues. However, this global “scaling up” of body size did not hold true for longitudinal growth of the skeleton because the tibias and radii of transgenic mice were identical in length to those of control mice. Currently, there is no known human condition that directly mirrors the tissue IGF-1 overexpression model, although patients with trisomy 15q26, and thus ubiquitous upregulation of the IGF-1R, also exhibit overgrowth and tall stature.(44–45)

The abovementioned studies indicate a common theme of growth inhibition and, in many cases, impaired skeletal development in the presence of a global GH/IGF-1 deficiency (Table 1). However, specific changes in body composition, bone size/shape, and timing of ossification are highly variable and appear to depend on sex, genetic background, age, and where along the GH/IGF-1 signaling pathway the disruption exists. To more specifically control for these variables, mouse models were created to tease apart the endocrine (serum) role of Igf-1 from its autocrine/paracrine (tissue) role.

**Table 1:** Mouse Models of the GH/Igf Axis and Their Human Counterparts

Target	Mutant name	Genetic background	Skeletal phenotype	Ref	Human counterpart (ref)
Global GH action	*Pit1*^*−/−*^ (*dw*/*dw*, Snell)	Mixed (from outbred)	GR, abnormal growth plate, decreased linear growth.	(2,72)	Mutation in *Pit1* locus affect multiple targets. Patients exhibit short stature (MIM No. 173110).
	*Prop1*^*−/−*^ (*dt*/*dt*, Ames)	Mixed (from outbred)	GR, reduced bone area and BMC	(73,5)	Mutation in the *Prop1* locus affect multiple targets. Patients exhibit short stature (MIM No. 601538).
	*Ghrhr*^*−/−*^ (*lit*/*lit*)	C57BL6	GR (60% of adult size), reduced cortical BMD, normal trabecular bone.	(74)	Isolated GHD type 1B (MIM No. 139191)
	*Ghrbp*^*−/−*^	C57BL6	GR (60% of adult size) reduced tibial length and decreased hight of growth plate	(75–76)	Laron syndrome (MIM No. 262500)
	*Stat5b*^*−*^*/*^*−*^	129 x BALB/c outcross	GR, impaired longitudinal growth, impaired endochondral ossification	(9,77)	GH insensitivity with immune deficiency (*Stat5b* gene deletion) (MIM No. 604260)
	*GH antagonist triglyceride*	C57BL/6J × SJL/J	GR (60% of adult size), fourfold increased body adiposity, reduced BMD	(78) and*	Not applicable
	*GH triglyceride*	C57BL/6J	GH overexpression increased body weight, tibial mass, and tibial density	(79)	Acromegaly (MIM No. 102200)/ gigantism, Sotos syndrome (MIM No. 117550)
Global IGF-1 action	*Igf1*^*−/−*^	CD-1	GR (30% of adult size), reduced cortical BMD, increased trabecular BMD	(16,80)	*Igf1* mutation(26,81)
		C57BL6			*Igf1* gene deletion(22)
		75% NMRI genetic background			(MIM No. 608747)
	*Igf1*^*m/m*^ (MIDI)	Not specified	GR, reduced femoral length and areal BMD	(82)	Not applicable
	Igf1^+/*−*^ (haploinsufficiency)	CD1	GR (70% of adult size), reduced femoral length and areal BMD	(18,19)	Not described in the literature
		MF1/DBA			
	*Igf1TG* (ubiquitous expression)	Not specified	Increased body weight and organ growth, normal skeletal size and morphology	(43)	Not applicable
	*Igf1r*^*−*^*/*^*−*^	129/Sv	Intrauterine GR, lethal (neonates at 45% of WT) delayed bone ossification	(24)	Not described in the literature
	*Igf1r* *+* */*^*−*^ (haploinsufficiency)	129/Sv	GR (90% of adult size)	(25)	IGF-1 resistance (MIM No. 147370)
	Inbred mouse lines	C3H	Serum and skeletal Igf-1 levels were greater in C3H mice which have a significantly larger femoral total area and cortical area as compared to C57BL/6 mice	(83)	Not applicable
		C57BL/6			
Global IGF-2 action	*IGF2*^*−/−*^	MF1 C57BL/6 Chimera	GR (60% of neonate size)	(84)	Not described in the literature
	*Igf2TG*	75% NMRI genetic background	Increased body weight, no effect on femoral architecture or BMD.	(80)	Not applicable
Global IGF axis	*Igf1*^*−/−*^ *Igf2*^*+/−*^ (haploinsufficiency)	MF1 C57BL/6 Chimera	GR (30% of adult size)	(24)	Not described in the literature
	*Igf1*^*−/−*^ Igf1r^*−*/*−*^		Lethal (neonates at 45% of WT)	(24)	Not described in the literature
	*Igf2*^*+/−*^ *Igf1r*^*+/−*^ (haploinsufficiency)		GR (30% of adult size)	(4)	Not described in the literature
	*Igf1*^*−/−*^ *GHR*^*−/−*^	Mixed Breeding (C57BL/6, 129Sv, MF!/DBA)	GR (17% of adult size)	(85)	Not described in the literature
Global IGF-1R mediators	*Irs1*^*−/−*^	CD-1 C57BL/6 Chimera	Growth retardation (50% of adult size); no delay in long bone ossification	(35)	Not described in the literature. Heterozygous mutation is associated with metabolic disorder.(86)
	*Irs1 sml*/*sml*	C3.SW-H2b/SnJ	Growth retardation (50% of adult size), low bone mineral density, reduced cortical and trabecular thickness, and low bone-formation rates	(36)	
	*Akt1/2*	MF1 C57BL/6 Chimera	Intrauterine GR, lethal (neonates at 45% of WT), delayed bone ossification	(87)	Not described in the literature
	*Foxo1/3/4* (conditional global deletion)	FVB/N C57BL6 mixed	Reduced BMD, significant reductions in trabecular bone	(88)	Not described in the literature
Global IGF bioavailability	*Igfbp1TG*	C57BL/6/CBA	GR, reduced skeletal mineralization	(39)	Not applicable
	*Igfbp2*^*−/−*^	C57BL6	Sex-related decrease in BMD (male)	(89)	Not described in the literature
	*Igfbp2 TG*	Not specified	Decreased (10%) carcass weight, reduced bone length, bone cross-sectional area, and BMC	(40,90)	Not applicable
	*Igfbp3*^*−*/*−*^	C57BL6	No effects on body weight or linear growth were noted	(91)	Not described in the literature
	*Igfbp3 TG*	CD-1	Reduced volumetric and cortical BMD, increased resorption	(41)	Not applicable
	*Igfbp4*^*−*/*−*^	C57BL/6	GR (85% of adult size)	(91)	Not described in the literature
	*Igfbp4 TG*	FVB/N	GR	(92)	Not applicable
	*Igfbp5*^*−/−*^	C57BL/6	No effects on body weight or linear growth were noted	(91)	
	*Igfbp5 TG*	C57BL/6JxCBA/CA	Sex-related decrease in BMD, impaired mineralization, decreased BFR	(42)	Not applicable
	*ALS*^*−/−*^	CD-1	GR (80% of adult size), reduced volumetric and cortical BMD, 10% reduction in femoral length	(93)	IGFALS deficiency (*ALS* gene deletion)
	C57BL/6				(MIM No. 601489)
	*ALS TG*	Balb C	Modest GR		
		CD-1		(38)	Not applicable
	*PappA*^*−/−*^ (IGFBP-4 protease)	C57BL/6 129Sv	GR (60% of adult size)	(94)	Not described in the literature
	*PappATG*	C57BL/6J XCBA/CA	Increased body weight	(95)	Not applicable
	*Igfbp3*^*−*^*/– -4*^*−/−*^ *-5*^*−/−*^	C57BL/6	GR (80% of adult size)	(91)	
	*Igfbp4*^*−*/*−*^; *PappA*^*−*/*−*^	129/C57BL/6	GR (90% of adult size)	(94)	
Endocrine IGF-1	*Liver-specific Igf-1 TG* (Hit)	C57BL/6	Liver-specific Igf-1 TG	(52–53,96–97)	
		FVB/N			
	*Liver-specific IGF1*^*−/−*^ (LID)	FVB/N	Normal growth, reduced volumetric and cortical BMD, 5% reduction in femoral length	(93)	
		C57BL/6			
	LID *ALS*^*−/−*^	FVB/N C57BL6 mixed	GR (70% of adult size), reduced cortical and trabecular BMD		
Autocrine/paracrine IGF-1 activity or bioavailbility	*Osteoblast-specific Igf1 TG*	FVB/N	Increased volumetric and cortical BMD	(64)	
	*Osteoblast-specific Igfbp4 TG*	FVB/N	Decreased in bone volume and cortical BMD	(61)	
	*Osteoblast-specific IGF1R*^*−/−*^	C57BL/6 x FVB/N	Normal growth, impaired mineralization	(58)	
	Osteoblast-specific *PappA TG*	C57BL/6J XCBA/CA	Increased calvarial BMD and tibial/femoral bone area and periosteal circumference	(98)	
	Chondrocyte-specific *IGF1*^*−/−*^	C57BL/6 X SJL	Body length, areal BMD, and BMC were reduced between 4 and 12 weeks	(56)	
	*Skeletal muscle/bone-specific IGF1*^*−/−*^ (collagen 1 and 2 expressing cells)	C567BL/6 X FVB/N	Reduced body weight, femoral BMD, femoral bone size, mineral apposition rate, and bone-formation rate.	(57)	
	*IGF1*^*−/−*^ *liver-specific Igf2 TG*	75% NMRI genetic background	GR, adults show similar body weight and length to the *Igf1*^*−/−*^ mice.	(80)	
Endocrine and autocrine/paracrine IGF-1 interplay	*Igf1*^*−/−*^ *liver-specific Igf1 TG* (KO-HIT)	FVB/N	Normalized skeletal growth and development due to threefold increase in endocrine (serum) Igf-1 levels.	(52–53)	
	*Igf1*^*−/−*^ *liver-specific Igf1 KI*	C57BL/6 CBA	Physiologic levels of liver-derived IGF-1 restored body size of the *Igf1* null mice to ∼70% of WT size	(65)	

* S Yakar personal note.

Mendelian Inheritance in Man = MIM; http://www.ncbi.nlm.nih.gov/omim/).

## IGF-1 as an Endocrine Factor

**Fig. 1:**
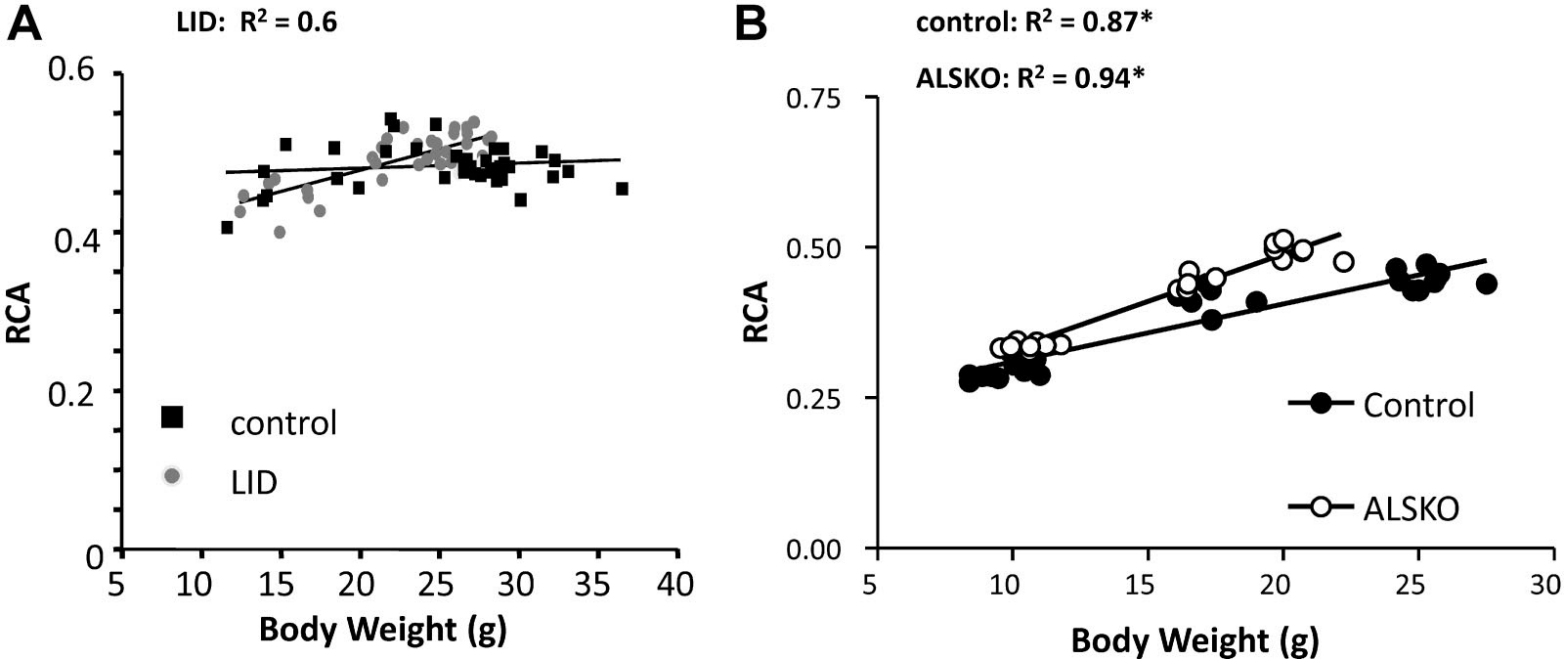
(*A*) Relative cortical area (RCA = Ct.Ar./Tt.Ar.) versus body weight for male LID and control mice during growth, as published previously.(49) (*B*) RCA versus body weight for male *ALS* KO and control mice during growth, as published previously.(50) For both LID and *ALS* KO mice, RCA is increased as body weight increases during growth compared with control mice, illustrating a small compensatory response of bone to decreased periosteal apposition.

Studies from inbred mouse strains with distant genetic backgrounds that have markedly different serum Igf-1 concentrations have reinforced the importance of serum Igf-1 for bone development.(46) The mouse strains with low Igf-1 (C57BL/6J) have reduced total BMD and cortical thickness, whereas mice with higher serum Igf-1 levels (C3H/HeJ) show increased total BMD and femoral cortical thickness. In addition, congenic mice (B6.C3H.6T) with a 40% reduction in serum Igf-1 also had reduced BMD and delayed development.(47) Given that the liver is responsible for nearly 75% of serum Igf-1 levels, ablation of *Igf1* gene expression in the liver was a promising approach to quantify the effects of serum Igf-1 on skeletal growth and development. Liver-specific Igf-1-deficient (LID) mice were created using the *Cre-LoxP* system. Preliminary data indicated an 80% decrease in serum Igf-1 levels, no change in body weight, and a small but significant decrease in body length from 3 to 8 weeks of age.(48) A subsequent study examined skeletal growth of LID mice in detail from 4 to 52 weeks of age and confirmed these initial findings while presenting new data on the role of Igf-1 in skeletal development after puberty.(49) Results of this study indicated that a constitutive loss (from birth) of serum Igf-1 resulted in significant reductions in body weight after puberty. Although no alterations in trabecular bone were found, significant decreases in femoral total area (Tt.Ar), cortical area (Ct.Ar), and polar moment of inertia (*J*_*o*_) were found beginning at 8 weeks of age. As a result, femurs were more slender (less robust) with reduced stiffness and reduced strength in bending. Thus reductions in serum Igf-1 tended to target cortical bone by preventing periosteal apposition during growth. Interestingly, marrow area (Ma.Ar) was not altered during early growth and actually decreased relative to controls from 16 to 32 weeks of age (endosteal infilling), suggesting that when serum Igf-1 levels are lowered early during growth, bones become more slender, but skeletal elements are able to activate a compensatory adaptive response by adding more bone endosteally to support increases in body weight. This was evident by an increase in relative cortical area (RCA) during growth (Fig. 1) such that the total amount of bone tissue present per total area of bone increased owing to endosteal infilling.

Serum Igf-1 levels also have been reduced in mouse models by altering the expression of one or more Igf-1-binding proteins. In *Igfbp3* knockout mice, serum Igf-1 levels were reduced by 40%, but body weight and length were increased by 8 weeks compared with controls.(48) Interestingly, these mice had decreased femoral trabecular bone volume fraction (BV/TV) and trabecular number (Tb.N) with no apparent changes in cortical bone size or tissue amount, suggesting that *Igfbp3* may have an Igf-1-dependent or -independent effects on the skeleton. The ALS is an important binding protein of Igf-1 and Igfbp-3 in serum, and when it is ablated in mice (*ALS* KO), it results in 65% reductions in serum Igf-1 levels, similar to the LID model. A detailed skeletal analysis of *ALS* KO mice from 4 to 16 weeks indicated reductions in body weight and body length throughout growth in both female and male mice.(50) In addition, by adulthood (16 weeks), *ALS* KO mice, both females and males, had reduced Tt.Ar., Ct.Ar., Ma.Ar, *J*_*o*_, and robustness (more slender). Similar to LID mice, *ALS* KO mice are also able to compensate for smaller, more slender bones through marrow infilling [reducing Ma.Ar and increasing their relative cortical area (RCA)]. Interestingly, the increased slenderness and compensatory increased marrow infilling (increased RCA) were more prominent in female than male *ALS* KO mice (Fig. 1). The data from male LID and *ALS* KO mice indicate a common role for serum Igf-1 in maintaining periosteal apposition during growth. The *ALS* KO mouse model is now of particular interest because human patients with ALS deficiency have been and continue to be reported in the literature. Although detailed quantification of skeletal structures in ALS-deficient patients has not been performed, a review of case reports has been published that indicates short stature and reduced BMD in a number of patients.(51)

Increased Igf-1 levels in serum were demonstrated in mice expressing hepatocyte-specific rat *Igf1* transgene (HIT). HIT mice exhibit two- to threefold increases in serum Igf-1 levels, which were accompanied by increases in body weight, body length, femoral length, and femoral Tt.Ar, Ct.Ar, Ct.Th, *J*_*o*_, and robustness.(52–53) Trabecular architecture also was examined in the HIT model, but only a few changes were observed at 16 weeks of age (Tb.Th increased in HIT mice). Thus the HIT phenotype is a scaling up of body size and cortical skeletal size with proportional changes in lean and fat mass. It should be noted that the HIT model is one of the few examples where Igf-1 alteration changed the composition of bone; HIT mice at 16 weeks were found to have a higher tissue mineral density (TMD) in both cortical and cancellous bone, indicating that more mineral was present in a given volume of bone than in control mice.

Together, studies of mice with reductions in serum Igf-1 revealed minor changes in body weight and length but significant decreases in transverse bone growth (Table 1). Reductions in serum Igf-1 during postnatal growth are extremely important in establishing bone robustness and suggest a possible role in determining increased fracture risk during adulthood and aging. On the other hand, increases in serum Igf-1 levels during growth lead to enhancement of all bone traits and may play a protective role later on during aging. They also emphasize the importance of serum Igf-1 in contributing to cortical size and bone density. Indeed, several papers have reported skeletal abnormalities in humans with low serum IGF-1.(54,55) In general, these have been patients with severe GH deficiency. These patients usually have normal volumetric BMD values and smaller bones. Increased fractures have been reported in some studies.(55) These studies are difficult to relate to the mouse models because, except for the case of *Igf1* gene deletion,(23) GH deficiency also has been present.

## IGF-1 as a Tissue Growth Factor

Tissue-specific regulation of IGF-1 is an important feature of many developmental processes. In the past 10 years, we have advanced our understanding of IGF-1 action on bone using cell-type specific IGF-1/IGF-1R inactivation. In a model where chondrocyte Igf-1 synthesis was disrupted, significant reductions in body weight, body length, total-body BMD, and femoral length were observed beginning at 4 weeks of age in both female and male mice.(56) Further, femoral width, as measured by periosteal circumference, was reduced in both sexes, although the differences were greater for males than for females. Conditional deletion of *Igf-1* in skeletal muscle and bone resulted in decreased femoral size, increased apoptosis, and decreased bone-formation rate.(57)

When the Igf-1R was disrupted in osteoblasts (Δ*Igfr* mice), no alterations in body size, weight, or femoral length by 6 weeks of age were found. However, Δ*Igfr* mice showed significant reductions in distal metaphyseal trabecular BV/TV, Tb.Th, TB.N, and MAR as well as increased osteoid volume and osteoid surface.(58) This study was crucial to establishing the role of Igf-1 in bone mineralization. Mice bearing *Igf1i* or *Igf1r* gene deletions also have been useful for determining if the known anabolic factors for bone require expression of Igf-1. Mice with an *Igf1r* depletion in osteoblasts showed decreased endosteal bone formation in response to PTH compared with controls. This defect was demonstrated in bone marrow stromal cells derived from these animals through a decreased number of alkaline phosphatase colonies and decreased mineralization in response to PTH.(59) These findings also have been replicated in *Igf1* knockout mice.(60) It should be noted that tissue-specific effects of Igf-1 have been hard to quantify because they are not easily separable from the endocrine reservoir of Igf-1. Thus, in the previous example of Δ*Igfr* mice, disruption of the Igf-1r will impair both autocrine/paracrine and endocrine Igf-1 effects on osteoblasts.

Tissue-specific expression of Igfbps leads to decreased Igf-1 bioavailability and generally shows phenotypes similar to the global *Igfbp* transgenics, namely, reduced body weight and length. Igfbp-4 overexpression in osteoblasts resulted in decreased femoral cortical density, cortical thickess, and periosteal circumference in both males and females by 6 weeks of age.(61) Mice expressing Igfbp-5 under the osteocalcin promoter demonstrated decreased BMD, trabecular bone volume, and bone formation,(62) as well as mineralization defects indicated by reduced mineral/matrix ratio in cortical bone and reduced collagen maturity in secondary ossification centers.(63) As was stated previously, local expression of Igfbps does not distinguish between autocrine/paracrine and endocrine Igf-1 effects but rather blocks the Igf-1 axis in a cell-specific manner. In contrast, Igf-1 overexpression in osteoblasts under the osteocalcin promoter (*OC-Igf1* transgenic mice) resulted in no change in serum Igf-1 levels or body weights up to 16 weeks but significant increases in cortical and trabecular BMD as well as trabecular bone volume and trabecular thickness.(64) In these *OC-Igf1* mice, histomorphometric parameters were no different from those of control mice at the later age (24 weeks), but increases in bone-formation rate were apparent at 6 weeks of age, indicating that increased bone accrual is accomplished largely during puberty.

As a point of comparison with the previously discussed HIT mouse mode, the KO-HIT mouse model lacks tissue Igf-1 in all tissues, and the sole source of Igf-1 production is through a transgene expressed in the liver. Thus serum Igf-1 levels are identical to HIT mice and threefold higher than control mice. Differences between HIT and KO-HIT mice therefore represent the consequences of tissue *Igf1* gene ablation from birth. Interestingly, body weight and length were not increased in KO-HIT mice, as they were in HIT mice, and morphologic analyses revealed that KO-HIT mice had skeletal properties similar to controls starting at 8 weeks of age.(52) However, early in development (before 8 weeks), significant reductions in femoral length, Tt.Ar, Ct.Ar, and robustness were evident. By 16 weeks, KO-HIT traits were normalized to control levels (eg, femoral length, Tt.Ar., and robustness) or exceeded control levels in a manner similar to HIT mice (ie, Ct.Ar). These findings are in agreement with another study in which liver Igf-1 was reexpressed in *Igf1* null mice resulting in a partial postnatal restoration of serum Igf-1 levels (∼50% of normal) and body weight (∼70% of normal).(65) Thus tissue Igf-1 appears crucial for early postnatal and pubertal development of cortical bone, but serum Igf-1 can permit “catchup” growth postpubertally (Table 1). Whether reexpression of normal levels of serum Igf-1 sufficient for normal growth in the absence of tissue Igf-1 remains unknown.

## Final Considerations

Throughout this article, the skeletal phenotypes of Igf-1 mouse models have been presented without context of their genetic backgrounds. This is an important consideration, and for each model discussed in this article, genetic backgrounds are given (where available) in Table 1. In addition, Table 1 offers a more extensive list of mouse models relating to the GH/Igf-1 axis as well as summaries of their skeletal phenotypes. Numerous studies indicate that differences in genetic background, whether complete(66,67) or even partial,(68–71) can result in significant alterations in skeletal properties and mechanical function. Therefore, any assessment of genetically engineered mice must consider the possibility that gene interactions from genetic background effects may determine at least partially the phenotype. As indicated in Table 1, the majority of Igf-1 mouse models have been created on a few select backgrounds (ie, CD-1, C57BL/6, and FVB/N) or on mixed backgrounds. Nevertheless, a review of the existing data from the different genetic backgrounds used indicates that the major roles of Igf-1 (eg, growth retardation from global deficiency, periosteal inhibition from reduced serum Igf-1 levels, etc.) exist despite differences in genetic background.

## Concluding Remarks

After 60 years of investigation, it is apparent that the GH/IGF axis plays a prominent regulatory role in skeletal development and mineral acquisition. Clinical studies as well as animal models have taught us that global loss of IGF-1 affects growth and skeletal gains at all ages, resulting in short stature and slender and weaker bones. With the development of a tissue-specific gene approach, we have advanced our knowledge regarding IGF-1's mode of action. We now know that loss of serum IGF-1 affects mainly postpubertal bone accrual. Longitudinal studies have demonstrated the importance of serum IGF-1 in transverse bone growth and periosteal bone apposition, as well as in bone adaptation to increases in body weight. We have learned that loss of tissue IGF-1 affects early postnatal and prepubertal growth, but there is some compensation when serum IGF-1 levels increase postpubertally. These studies also clearly demonstrate that the IGF-1 axis in osteoblasts is a strong determinant of bone mineralization.

## Future Directions

Notwithstanding our extensive knowledge of IGF-I action on the growing skeleton, its role in skeletal homeostasis during aging is still unclear. Moreover, our understanding of IGF-1 interactions with steroid hormones, insulin, PTH, sclerostin, and the Wnt pathway and their effects on the skeleton during growth and aging is incomplete. Other open questions regarding IGF-1 action include its role in osteocyte function and in osteoclastogenesis. Lastly, perhaps the most important question is: *How* and *when* should we intervene through manipulation of the GH/IGF axis to obtain a more robust, mechanically fit skeleton.
